# Pathobiological analysis of African swine fever virus contact-exposed pigs and estimation of the basic reproduction number of the virus in Vietnam

**DOI:** 10.1186/s40813-023-00330-0

**Published:** 2023-06-29

**Authors:** Sang-Ik Oh, Ngoc Anh Bui, Vuong Nghia Bui, Duy Tung Dao, Ara Cho, Han Gyu Lee, Young-Hun Jung, Yoon Jung Do, Eunju Kim, Eun-Yeong Bok, Tai-Young Hur, Hu Suk Lee

**Affiliations:** 1 Division of Animal Diseases & Health, Rural Development Administration, National Institute of Animal Science, Wanju, 55365 Republic of Korea; 2 Bio-Safety Research Institute and Laboratory of Veterinary Pathology, College of Veterinary Medicine, Jeonbuk National University, Iksan, 54596 Republic of Korea; 3 Virology Department, National Institute of Veterinary Research, Hanoi, Vietnam; 4 International Livestock Research Institute, Hanoi, Vietnam; 5 College of Veterinary Medicine, Chungnam National University, Daejeon, 34134 Republic of Korea

**Keywords:** African swine fever, African swine fever virus, Basic reproduction number (R_0_), Pathobiology, Transmission rate (β), Vietnam

## Abstract

**Background:**

African swine fever (ASF), caused by African swine fever virus (ASFV), is a fatal disease affecting wild and domestic pigs. Since China reported the first ASF outbreak in August 2018, ASFV has swept over the neighbouring Asian countries. However, studies involving experimental pig-to-pig ASFV transmission in Vietnam are lacking. The main objective of this experimental study was to demonstrate the pathobiological characteristics of ASFV contact-exposed pigs and estimate their basic reproduction number (R_0_) in Vietnam. Fifteen pigs were randomly divided into two groups: experimental (n = 10) and negative control (n = 5) groups. One pig in the experimental group was intramuscularly inoculated with ASFV strain from Vietnam in 2020 and housed with the uninoculated pigs during the study period (28 days).

**Results:**

The inoculated pig died 6 days post-inoculation, and the final survival rate was 90.0%. We started observing viremia and excretion of ASFV 10 days post-exposure in contact-exposed pigs. Unlike the surviving and negative control pigs, all necropsied pigs showed severe congestive splenomegaly and moderate-to-severe haemorrhagic lesions in the lymph nodes. The surviving pig presented with mild haemorrhagic lesions in the spleen and kidneys. We used Susceptible-Infectious-Removed models for estimating R_0_. The R_0_ values for exponential growth (EG) and maximum likelihood (ML) were calculated to be 2.916 and 4.015, respectively. In addition, the transmission rates (β) were estimated to be 0.729 (95% confidence interval [CI]: 0.379–1.765) for EG and 1.004 (95% CI: 0.283–2.450) for ML.

**Conclusions:**

This study revealed pathobiological and epidemiological information in about pig-to-pig ASFV transmission. Our findings suggested that culling infected herds within a brief period of time may mitigate the spread of ASF outbreaks.

**Supplementary Information:**

The online version contains supplementary material available at 10.1186/s40813-023-00330-0.

## Background

African swine fever virus (ASFV) is a large double-stranded DNA virus of the *Asfarviridae* family and *Asfivirus* genus and is a highly contagious pathogen in pigs [1]. African swine fever (ASF), one of the most important transboundary swine diseases, has a serious economic impact on the global pig industry and threatens food security. Mortality rates in domestic and most wild pigs infected with ASFV can reach up to 100% [2, 3].

ASF outbreaks have recently been reported in Asia, with the first case reported in August 2018 in China [4] and then spreading to other Asian countries [5, 6]. In Vietnam, the first case was reported in February 2019 in Hung Yen province, 50 km from Hanoi and 250 km from the Chinese border [6]. Since then, ASF outbreaks have been detected in all 63 provinces of Vietnam, and 20–25% (6–6.15 million) of the pig population has perished due to ASF and massive depopulation policies [7]. One of the main risk factors is poor biosecurity and use of food waste as pig feed in small-scale farms, which account for 60–65% of pig production in Vietnam [8]. In the past 5 years, several in vivo experiments have been performed to evaluate the pathogenicity of ASFV isolated from Asia [9–13]. Our previous study revealed that ASFV isolated from Vietnam induced peracute to acute forms of the disease, resulting in high mortality (100% death within 8 days post-inoculation [dpi]) with a short incubation time (3.7 ± 0.5 dpi) [14].

The basic reproduction number (R_0_) is an important parameter for describing the transmissibility of infectious diseases in a population and is useful for better understanding the characteristics of pathogens [15]. R_0_ was defined as the expected number of secondary infections from an infectious individual in a completely susceptible population [16]. It is affected by various biological and environmental factors as well as social behaviours, which are estimated using various complex mathematical models [17]. If R_0_ is greater than 1, the pathogen will continue to propagate in the susceptible population. The disease will decline and eventually fade out if R_0_ is less than 1.

In Vietnam, very few studies have calculated R_0_ at the farm level [18, 19]. Additionally, to the best of our knowledge, no studies have estimated R_0_ for ASF using experimental pig-to-pig transmission in Vietnam. Therefore, the primary objective of this study was to investigate the clinical signs and pathological lesions in ASFV contact-exposed pigs and to estimate R_0_ for the first time using an experimental study.

## Results

### Clinical assessment and pathological lesions

**Fig. 1:**
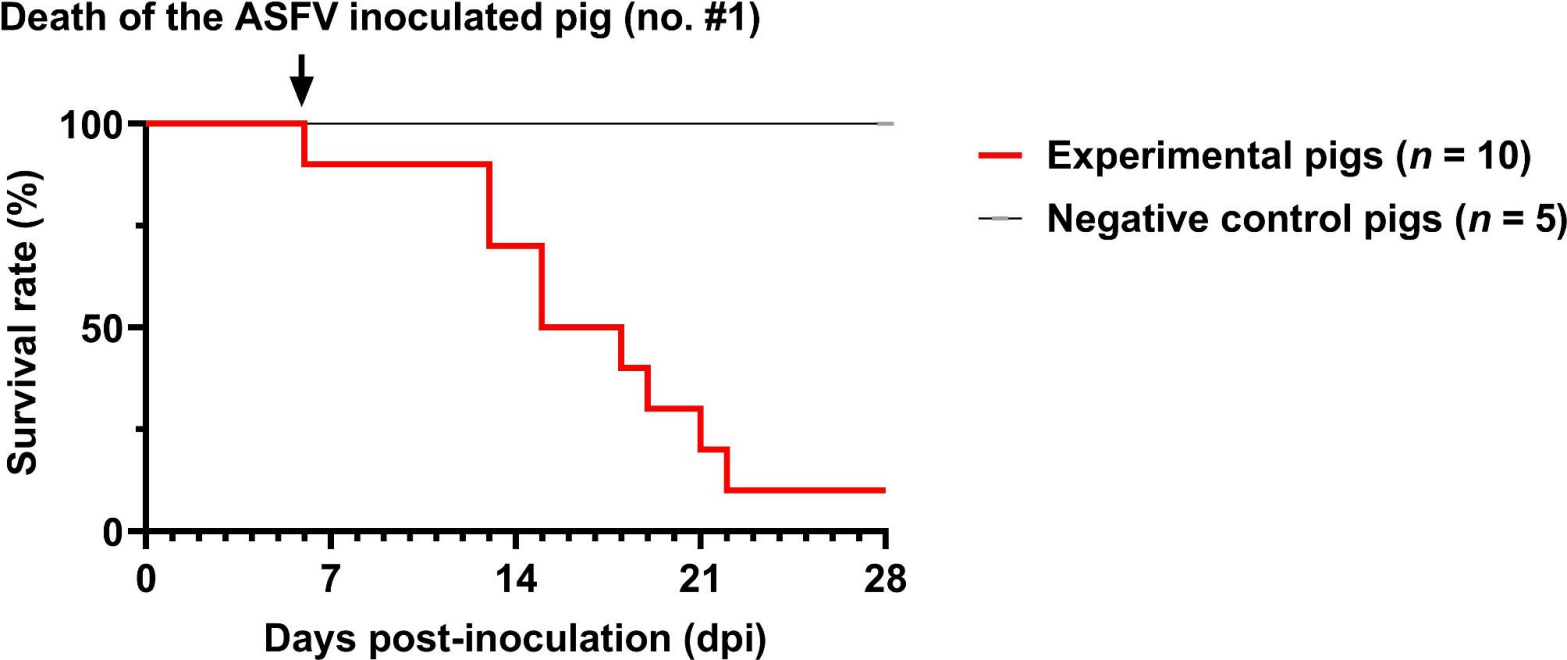
Survival rate of the experimental pigs. Survival rate in the pen with the experimental group [red line; African swine fever virus-infected pig (*n* = 1) and direct contact pigs (*n* = 9)] and negative control group (black line; *n* = 5)

**Fig. 2:**
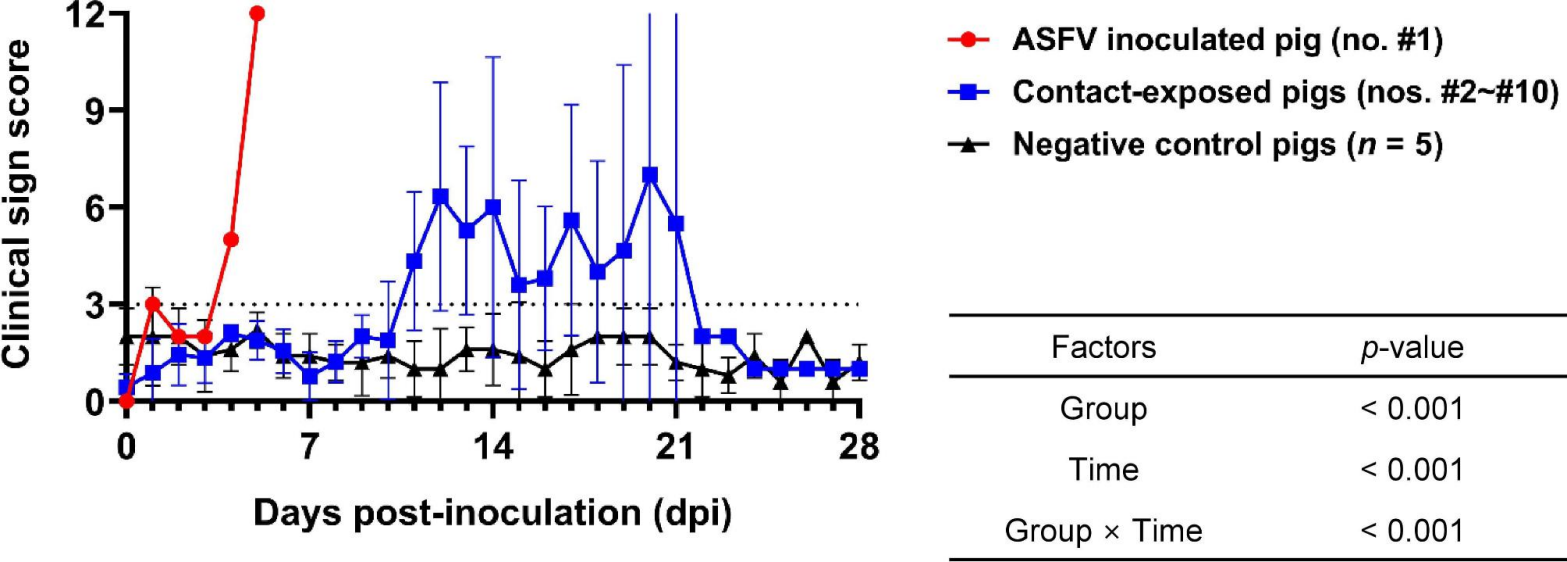
Average clinical sign scores in the experimental pigs. Red circles and horizontal line, African swine fever virus-inoculated pig; blue squares and horizontal line, direct contact-exposed; black triangles and horizontal line, negative control pigs

The survival rate (90%) and onset of death in the experimental pigs are shown in Fig. 1. Experimental inoculation of the pig (no. #1) with ASFV was performed, and the animal was euthanised at 6 dpi. The first death in the contact group occurred at 13 days post-exposure (dpe). Pig no. #10 survived for the entire experimental period (up to 28 dpe). Excluding the surviving pig (no. #10), the average death period in the contact group was 16.9 ± 3.1 dpe. Clinical sign scores and rectal temperatures in the experimental group varied throughout the experimental period (Table 1). The average clinical sign scores between the contact and negative control groups were significantly different (*p* < 0.001), and intergroup comparisons also showed a significant difference between group and over time (*p* < 0.001) (Fig. 2). All necropsied pigs, except the surviving and negative control pigs, showed severe congestive splenomegaly and moderate-to-severe haemorrhagic lesions in the lymph nodes (Table 2). However, the surviving pig (no. #10) only presented with mild haemorrhagic lesions in the spleen and kidneys. Haematoxylin and eosin-stained spleens showed moderate-to-severe lymphoid depletion (nos. #1 and #5) and follicular atrophy (nos. #3 and #7), whereas such histopathological lesions were not observed in the surviving pig (no. #10) and the negative control (no. #15) (See Supplementary Fig. 1, Additional File 1). In addition, severe and diffuse engorgement of the red pulp of the spleen was observed in two contact pigs (nos. #5 and #7), whereas a mild engorgement lesion was detected in the surviving pig (no. #10).

**Table 1:** Time-serial changes in rectal temperature and clinical sign scores in experimental pigs

Dpi	Clinical sign scores (rectal temperature, °C) in pig no.^*^									
	#1	#2	#3	#4	#5	#6	#7	#8	#9	#10
0	0 (38.4)	0 (38.4)	0 (39.1)	0 (39.8)	0 (38.6)	1 (39.6)	1 (39.6)	1 (39.6)	0 (39.1)	1 (39.0)
1	3 (39.6)	2 (39.5)	0 (39.1)	0 (39.3)	0 (39.3)	3 (39.6)	0 (39.3)	0 (39.4)	0 (39.3)	3 (39.6)
2	2 (40.2)	2 (39.6)	1 (39.1)	0 (39.3)	2 (39.5)	3 (40.0)	2 (39.7)	0 (39.0)	0 (39.4)	3 (39.6)
3	2 (40.2)	2 (39.6)	2 (39.6)	2 (39.9)	0 (39.3)	0 (39.4)	2 (39.8)	2 (39.7)	0 (39.1)	2 (39.7)
4	**5 (40.8)**	2 (40.1)	2 (39.9)	3 (39.7)	2 (39.5)	2 (40.2)	2 (40.2)	2 (39.9)	2 (39.6)	2 (39.8)
5	**12 (41.0)**	2 (40.3)	3 (39.8)	2 (39.8)	2 (39.7)	2 (39.7)	2 (39.7)	2 (39.8)	0 (39.4)	2 (39.7)
6	E	2 (39.8)	0 (39.3)	2 (39.8)	2 (39.8)	2 (40.1)	2 (39.8)	0 (39.4)	2 (40.0)	2 (39.7)
7		2 (39.6)	0 (39.4)	0 (39.3)	0 (39.4)	0 (39.2)	1 (39.5)	2 (39.8)	0 (39.4)	2 (39.6)
8		0 (39.4)	1 (39.0)	0 (39.1)	1 (39.4)	2 (39.7)	1 (39.4)	2 (39.8)	2 (39.6)	2 (39.5)
9		0 (39.3)	2 (39.5)	2 (39.7)	3 (40.4)	2 (39.9)	3 (39.9)	2 (39.8)	2 (39.5)	2 (39.6)
10		0 (39.3)	0 (39.4)	2 (40.1)	**7 (41.6)**	2 (40.2)	0 (39.4)	2 (39.6)	0 (39.0)	**4 (40.6)**
11		2 (39.8)	0 (39.3)	**6 (41.5)**	**7 (41.3)**	**8 (41.4)**	3 (39.8)	**7 (41.6)**	2 (39.6)	**4 (40.6)**
12		**6 (41.7)**	2 (39.8)	**6 (41.3)**	**15 (41.7)**	**10 (42.0)**	2 (39.9)	**10 (41.5)**	1 (39.4)	**5 (40.6)**
13		**7 (42.3)**	3 (39.7)	**8 (41.8)**	D	**9 (42.2)**	2 (40.0)	D	2 (40.1)	**5 (40.7)**
14		**6 (41.8)**	2 (39.9)	**12 (42.0)**		**14 (41.5)**	2 (39.5)		2 (39.5)	**4 (40.7)**
15		**8 (41.8)**	2 (39.9)	D		D	2 (40.2)		2 (39.5)	**4 (41.0)**
16		**6 (41.8)**	2 (39.5)				**5 (41.4)**		2 (39.7)	**4 (40.7)**
17		**10 (41.5)**	**5 (39.7)**				**5 (41.3)**		2 (39.6)	**6 (40.5)**
18		E	**4 (40.0)**				**7 (41.2)**		2 (40.2)	3 (40.2)
19			**6 (41.3)**				D		**6 (41.7)**	2 (40.5)
20			**8 (41.4)**						**11 (41.9)**	2 (40.1)
21			**9 (41.8)**						E	2 (40.3)
22			D							2 (40.2)
23										2 (40.3)
24										1 (39.4)
25										1 (39.2)
26										2 (39.7)
27										1 (39.0)
28										1 (39.0)
29										E

*, Pig no. #1, inoculated with African swine fever virus; pig nos. #2–10, pigs with within-pen direct contact. Bold text indicates that the pig showed clinical signs of African swine fever virus infection (> 3 scores). Dpi, day post-inoculation; E, euthanasia; D, death

**Table 2:** Presence of major gross pathological lesions in experimental pigs

Major gross lesions	Pig no.^*^ (death period)					
	#1 (6 dpi)	#3 (22 dpi)	#5 (12 dpi)	#7 (19 dpi)	#10 (Survive)	#15 (NC)
Congestive splenomegaly	+++	+++	+++	+++	+	-
Haemorrhagic enlargement of submandibular LN	+++	+++	++	++	-	-
Haemorrhagic enlargement of mesenteric LN	+++	+++	++	+++	-	-
Hyperaemia of tonsil	+	-	-	+	-	-
Hyperaemia of lung	+	+++	-	**+++**		-
Petechiae or haemorrhages in the liver	+	+	-	++	-	-
Petechiae or haemorrhages in the kidney	+++	+++	+	++	+	-
Petechiae or haemorrhages in the intestine	+++	++	+	-	-	-
Petechiae or haemorrhages in the heart	-	+++	++	+	-	-
Abdomen exudative fluid	+	++	+	++	-	-
Skin erythema	+	-	++	-	-	-

^*^Pig no. #1, ASFV-inoculated pig; pig nos. #2, #3, #5, #7, and #10, within-pen direct contact pigs; pig no. #15, negative control pig. LN: lymph node, NC: negative control, -: no lesion, +: mild, ++: moderate, +++: severe

### Onset of virus infection

**Fig. 3:**
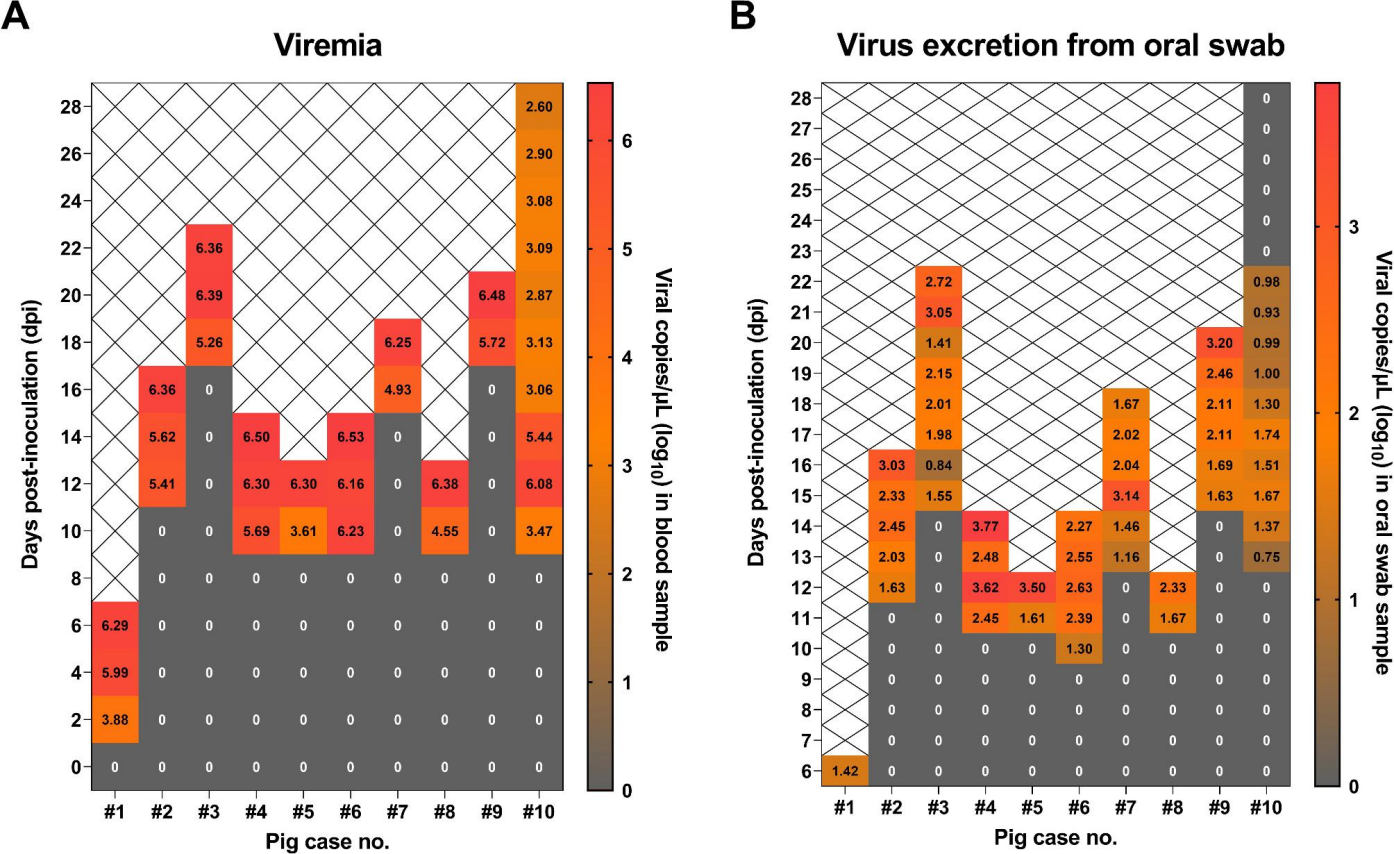
Patterns of African swine fever virus (ASFV) detection in pigs (*n* = 10) with experimental pig-to-pig infection. One ASFV-inoculated pig (no. #1) and nine direct contact-exposed pigs within pen (no. #2–#10). (**A**) Viral copies/µL from individual blood sample from experimental pigs. (**B**) Viral copies/µL from individual oral swab sample from experimental pigs

Time-dependent serial changes in viral load in blood samples from each experimental pig are shown in Fig. 3a. ASFV DNA was detected in blood samples from pig no. #1 at 2 dpi (7.6 × 10^3^ copies/µL), 4 dpi (9.9 × 10^5^ copies/µL), and 6 dpi (1.9 × 10^6^ copies/µL). Five pigs (55.5% of direct contact pigs) started to develop viremia from 10 dpe. The average onset time of viremia in the contact group was 12.7 ± 3.4 dpe. Notably, viremia was detected in the surviving pigs (no. #10) until the end of the experiment (28 d). Viral load in oral swab samples from pig no. #1 was 2.7 × 10^1^ copies/µL (Fig. 3b) and those from nasal and rectal swabs were 1.0 × 10^4^ and 6.1 × 10^3^ copies/µL, respectively (See Supplementary Fig. 2, Additional File 1). The average onset time of virus excretion from oral swab samples in the contact group was 12.3 ± 1.7 dpe. The mean onset times of virus detection in nasal and rectal swab samples from the contact group were 11.7 ± 1.3 and 13.9 ± 2.9 dpe, respectively. ASFV DNA was detected in oral samples from the surviving pig (no. #10) from days 13 to 22. These results indicate that the virus contact-exposed pigs were infected during 10–15 dpe (average 11.7 ± 2.1 dpi).

### Estimation of transmission parameters

For the first inoculated pig, the infectious period (T) was estimated to be 4 days as the virus was first isolated at 2 dpi and death occurred at 6 dpi. The mean and standard deviation of the generation time (GT) were estimated to be 1.039 and 0.845 days, respectively, using a log-normal distribution (Table 3). The R_0_ values for exponential growth (EG) and maximum likelihood (ML) were 2.916 (95% confidence interval [CI]: 1.516–7.059) and 4.015 (95% CI: 1.131–9.801), respectively. In addition, the transmission rates (β) were estimated to be 0.729 (95% CI: 0.379–1.765) for EG and 1.004 (95% CI: 0.283–2.450) for ML.

**Table 3:** Estimated parameters from the models

Parameters	Description	Exponential growth (EG) method	Maximum likelihood (ML) method
R_0_	Basic reproduction number	2.916 (95% CI: 1.516–7.059)	4.015 (95% CI: 1.131–9.801)
T	Infectious period	4	4
γ	Removed rate	0.25	0.25
β	Transmission rate	0.729 (95% CI: 0.379–1.765)	1.004 (95% CI: 0.283–2.450)

CI, confidence interval

## Discussion

The main route of ASFV transmission is direct contact between infectious and susceptible pigs [20]. Therefore, it is necessary to establish the clinical characteristics and transmission rates of contact-exposed pigs for ASF prevention and control. In particular, the Vietnamese government officially changed the ASF control policy with the option of partial culling on outbreak farms in July 2020 [21]. Therefore, this study aimed to elucidate R_0_ of ASFV in an experimental environment (ASFV introduction in viral-free herds) with detailed clinical information on contact-exposed pigs.

This study showed that the onset of ASFV excretion in contact-exposed pigs was 10 dpe, which was later than that reported in previous studies in China (6 dpe) and Europe (7.6 ± 2.6 dpe) [12, 22]. Moreover, these studies reported that the ASFV DNA from contact-exposed pigs was detected before the death of inoculated pigs (7–9 and 9 dpi, respectively) [12, 22]. In this study, the first viral infection in contact-exposed pigs (10 dpe) was detected 4 days after the death of the ASFV-inoculated pig (6 dpi). The discrepancy in the time taken to detect infection in ASFV-inoculated pigs could be explained by the differences in the number of inoculated pigs and overall sample size [12, 22]. The first clinical signs of ASFV infection in the contact group emerged at 10 dpe, suggesting that the time of the first death in ASF-infected pigs was 96 h, which is a reasonable time to prevent ASFV transmission within herds and in farms. Therefore, if partial culling could be performed within 96 h of the first ASFV diagnosis in farms, further spread of ASFV may be prevented. However, there remain many obstacles to establishing an effective partial culling policy. In particular, it is essential to consistently apply the methods for early detection of ASF in non-culling pig herds. Undiagnosed non-culling pigs or survivors in outbreak farms could serve as silent carriers of ASFV.

We previously reported that the detection of ASFV genomic DNA in nasal and oral swabs (3 dpi) was later than that in blood samples (1 dpi) of intramuscularly inoculated pigs [14]. However, viral DNA in this study was observed in oral samples from three contact-exposed pigs (nos. #3, #7, and #9) before the animals developed viremia. These differences in viral excretion patterns could be attributed to the fact that ASFV is known to first replicate in monocytes/macrophages in the lymph nodes close to the initial site of infection [23]. The most important ASFV transmission routes are ingestion of virus-contaminated feed, drinking contaminated water, and swallowing of virus particles from infected pigs [24]. As such, ASFV may replicate in the submandibular lymph nodes in contact-exposed pigs. Therefore, oral sampling or rope-based oral fluid collection [14] is a more reliable method for early detection in ASFV contact-exposed pigs than blood sampling.

Although the highly virulent ASFV responsible for the Asian ASF epidemic is known to cause 100% mortality in infected pigs, we found that 10% of pigs (one out of 10 pigs) in the experimental herd survived. Previous studies have highlighted the importance of the survivors and convalescent pigs, which might become carriers of ASFV [25, 26]; however, few studies have experimentally assessed the pathobiological characteristics of surviving pigs with Asian-epidemic ASFV infection. The surviving pig (no. #10) showed viremia from 10 dpe until the end of the experiment (28 dpe); however, ASFV genomic DNA was not detected in oronasal samples after 24 dpe. Moreover, ASFV antibody positivity was observed in the surviving pig from 26 dpe until euthanasia (data not shown). Although a long-term experiment is needed to elucidate the role of ASFV survivors, the present findings suggest that pigs surviving infection with a highly virulent ASFV strain would not act as carriers after convalescence. Our finding was consistent with that of a previous study on moderately virulent ASFV in Europe, with long-term monitoring [27]. The gross lesions of contact pigs (except the surviving pig) showed severe congestive splenomegaly, haemorrhagic enlargement of lymph nodes, petechial lesions in the kidneys, and the presence of abdominal exudative fluid, which are commonly observed in ASF-infected pigs [9, 10, 12, 20]. However, the surviving pig (no. #10) was intact, except for mild haemorrhagic lesions in the spleen and kidneys. Histopathological lesions in the spleens of contact-exposed pigs showed moderate-to-severe lymphoid depletion, atrophy of follicles, and engorgement; however, mild haemorrhagic lesions were observed in the survivor. Although a recent study investigated the pathological lesions of the survivors (qPCR-negative in blood and oral samples) in farms with ASF outbreak [28], the present study is the first to evaluate the pathomorphological lesions in the surviving pig (ASFV presence in the blood, but not in oral samples) by exposing the animals to the highly virulent genotype II ASFV.

We found that the estimated infectious period from the first case was approximately 4 days, which is consistent with the findings of previous studies (3–4 days) [22, 29, 30]. However, one experimental study suggested that the minimum infectious period was 6–7 days, whereas the maximum was between 20 and 40 days [31]. In addition, the transmission rate parameters (β) using the EG and ML methods were estimated to be 0.729 (95% CI: 0.379–1.765) and 1.004 (95% CI: 0.283–2.450), respectively. These values are slightly similar to those of an experimental study (0.6, 95% 0.3–1.0 per day) conducted in the UK [30] and higher than those of a recent farm investigation study (less than 0.37) conducted in Vietnam [19].

Our estimated R_0_ values were slightly higher or similar to those reported in previous studies in domestic pigs in the UK (R_0_: 2.8, 95% CI 1.3–4.8) and Uganda (R_0_: 1.58–3.24) [30, 32], while some studies reported higher values in China (R_0_: 4.83–11.90) [33] and in the Netherlands (R_0_: 4.9–66.3) [31]. These direct comparisons of R_0_ among studies have certain limitations, as the study designs and environmental conditions are different and R_0_ can be affected by various environmental factors [17, 20, 34]. For instance, it can be largely dependent on the pig species, pig population, and virulence of the ASFV isolates or strains.

The susceptible-infection-related (SIR) model was used to calculate R_0_, β, and γ, which are useful for evaluating cost-effective control and prevention measures in the Vietnamese context. In Vietnam, large-scale farms (accounting for less than 10% of pig production) have a better biosecurity system, whereas small- and medium-sized farms are the main source of ASFV infection because of poor biosecurity. Previous studies in Vietnam have suggested that ASF transmission can be reduced by applying strict movement controls and biosecurity [8, 35]. It was assumed that indirect contact (e.g., use of food waste and movement) contributed to more than 70% of ASF transmission in small-scale farms in Vietnam [36]. Although the authorities have already prohibited the use of kitchen waste or swill for domestic pigs since the first ASF outbreak, it is still widely practiced by small-scale pig farmers. In addition, it is well known that wild boars and soft ticks could be the main sources of infection in several countries [37–39]. In Asia, wild boars with ASF have been reported in China, India, Malaysia, and South Korea [6, 33, 40]; however, no study has been conducted to evaluate the possible roles of wild boars in the spread of viruses to domestic pigs in Vietnam. Therefore, it is necessary to conduct epidemiological investigations into the transmission route of the ASF virus.

Two techniques (EG and ML) were used to estimate R_0_ in our experimental study. The ML method showed a higher R_0_, but it was not significantly different from the EG method. The recent study in Vietnam conducted by Mai et al. [19] calculated R_0_ as 1.66 and 1.40 depending on the farm scale, which were lesser than our estimations of R_0_. It has already been shown that R_0_ can be influenced by environmental factors and modelling approaches [17]. Moreover, the previous study used real field data (farm scale: 100–999), which were more likely to be affected by other environmental factors (e.g. farmers’ behaviour, awareness, and control policies). R_0_ is useful for evaluating the effectiveness of disease control measures. For instance, if R_0_ is less than 1, an infectious disease fades out in a population. In addition, it can be used to estimate herd immunity (formula: 1 - $\frac{1}{R0}$), which is defined as the majority of a population developing immunity against an infectious disease, either through vaccination or due to a previous infection [41].

## Conclusions

This is the first experimental study on the transmission of ASFV in a population with detailed pathobiological information and the first to estimate R_0_ in Vietnam. Our results indicate that the virus began to spread by contact-exposed pigs 10 dpe after the death of the first inoculated pig (6 dpi) within the same pen. This result suggests that culling infected herds (pens) on an identical farm within a short period of time could lessen the impact of the ASF outbreak. In addition, our transmission experiment demonstrated the possibility of survival in contact-exposed pigs with intact-to-mild pathological lesions. Although the survivor did not exhibit clinical signs and excreted ASFV from oral, nasal, and faecal sources, further long-term studies are needed to clarify the risk of retransmission by the surviving pigs. The R_0_ values of ASFV were estimated to be 2.916 (EG) and 4.015 (ML), indicating that the virus is contagious in a pig herd. Since a vaccine is not available, the early detection of ASFV-infected pigs is important, and enhanced biosecurity measures should be applied by small-scale farmers to minimise the risk of transmission to domestic pigs.

## Methods

### Animal experiments

The ASFV strain used in this study was obtained from pig’s blood collected during an ASF outbreak farm in 2020 in Thanh Hóa province, Vietnam (GenBank accession no. OP615344). The virus was cultured and quantified as previously described [14]. Fifteen healthy, 6-week-old pigs (Yorkshire × Landrace × Duroc) were obtained from the same herd on a commercial pig farm in Vietnam. All pigs were tested and confirmed to be seronegative for endemic pathogens in Vietnam, including foot-and-mouth disease virus, porcine respiratory and reproductive syndrome virus, classical swine fever virus, porcine circovirus 2, ASFV, and Mycoplasma *spp*. The pigs were randomly divided into two groups: an experimental group (*n* = 10) and a negative control group (*n* = 5). To investigate the pathobiological characteristics in ASFV contact-exposed pigs and estimate R_0_, one pig (no. #1) in the experimental group was intramuscularly inoculated with 1 mL of the 10^3.5^ 50% hemadsorption dose (HAD_50_/mL) ASFV and housed with non-inoculated pigs (nos. #2–#10; contact group). The pigs were euthanised according to endpoint criteria described previously [42].

### Sampling and clinical assessment

The daily clinical signs and rectal temperatures of all pigs were recorded until the end of the experiment (28 dpe). Clinical sign scores were estimated and calculated based on a previous study [14]. Every 2 days, blood and daily swab (oral, nasal, and rectal) samples were collected from individual pigs to detect ASFV DNA via quantitative PCR (qPCR) using a VDx ASFV qPCR kit (Median Diagnostics, Chuncheon, South Korea). Necropsies were performed on five representative pigs (nos. #1, #3, #5, #7, and #10) in the experimental group, and one in the control group (no. #15). Gross and histopathological lesions (spleen) were analyzed as described in our previous study [9]. To evaluate the time series changes in clinical sign scores between the contact group (n = 9) and the negative control group (n = 5), a linear mixed effect model with repeated measures was used by SPSS version 26.0 (IBM, Armonk, NY, USA).

### Estimation of the basic reproduction number (R0) and transmission rate (β)

R_0_ was defined as the average number of secondary cases caused by a single infection in a susceptible population. We used SIR models to estimate R_0_ from our transmission experiment. A susceptible animal (*S*) becomes infectious (*I*) and is then removed (*R*) by depopulation or death at time *t*. The main assumption is that the entire population at the beginning is susceptible, which can be described by an equation based on time (*t*):

$$\frac{dS}{dt}= \frac{-\beta SI}{N}$$

$$\frac{dI}{dt}=\frac{\beta SI}{N}-\gamma I$$

$$\frac{dR}{dt}=\gamma I$$

In the equation model, β is the transmission rate, which is the probability of disease transmission between susceptible and infectious individuals, while γ is the removal rate. Based on the calculation of R_0_, the transmission rate (β), the daily rate that infectious cases cause new cases in a population, was calculated using the following formula [43]:

$$\beta =\frac{{R}_{0}}{Infectious period \left(T\right)}$$

In our study, the infectious period was estimated to be 4 days. The best-fitting GT distribution for a series of serial intervals was estimated using the est.GT function in the R0 package in R version 4.2.1. Subsequently, the mean and standard deviation of GT were calculated. Next, we constructed EG and ML models to estimate R_0_ with 95% CI. R_0_ can be estimated at different times during an epidemic and several methods have been proposed by researchers [44, 45]. In our study, we employed two methods: EG and ML. EG was summarised by Wallinga et al. (2007) [44] and they stated that the EG rate during the early phase of an outbreak can be linked to the initial reproduction ratio. ML estimation was proposed by White et al. (2009) [45] and they stated that the number of secondary cases caused by an index case exhibits a Poisson distribution with an expected value of R_0_.

## Electronic supplementary material

Below is the link to the electronic supplementary material.

Supplementary Material 1

## Data Availability

The data presented in this study are available on request from the first and corresponding authors on reasonable request.

## Abbreviations

ASF: African swine fever
ASFV: African swine fever virus
R_0_: Basic reproduction number
EG: Exponential growth
GT: Generation time
HAD50/mL: 50% hemadsorption dos
T: Infectious period
ML: Maximum likelihood
qPCR: Quantitative PCR
SIR: Susceptible-infection-related
β: Transmission rate
