# Supplementary material for: Pathobiological analysis of African swine fever virus contact-exposed pigs and estimation of the basic reproduction number of the virus in Vietnam
Source: Porcine Health Manag. 2023 Jun 29;9:30. doi: 10.1186/s40813-023-00330-0 (PMC10311738; doi:10.1186/s40813-023-00330-0)
Supplement: Supplementary file 1 — Supplementary Material 1 [file 40813_2023_330_MOESM1_ESM.docx]

## Additional File 1

## Supplementary Figures


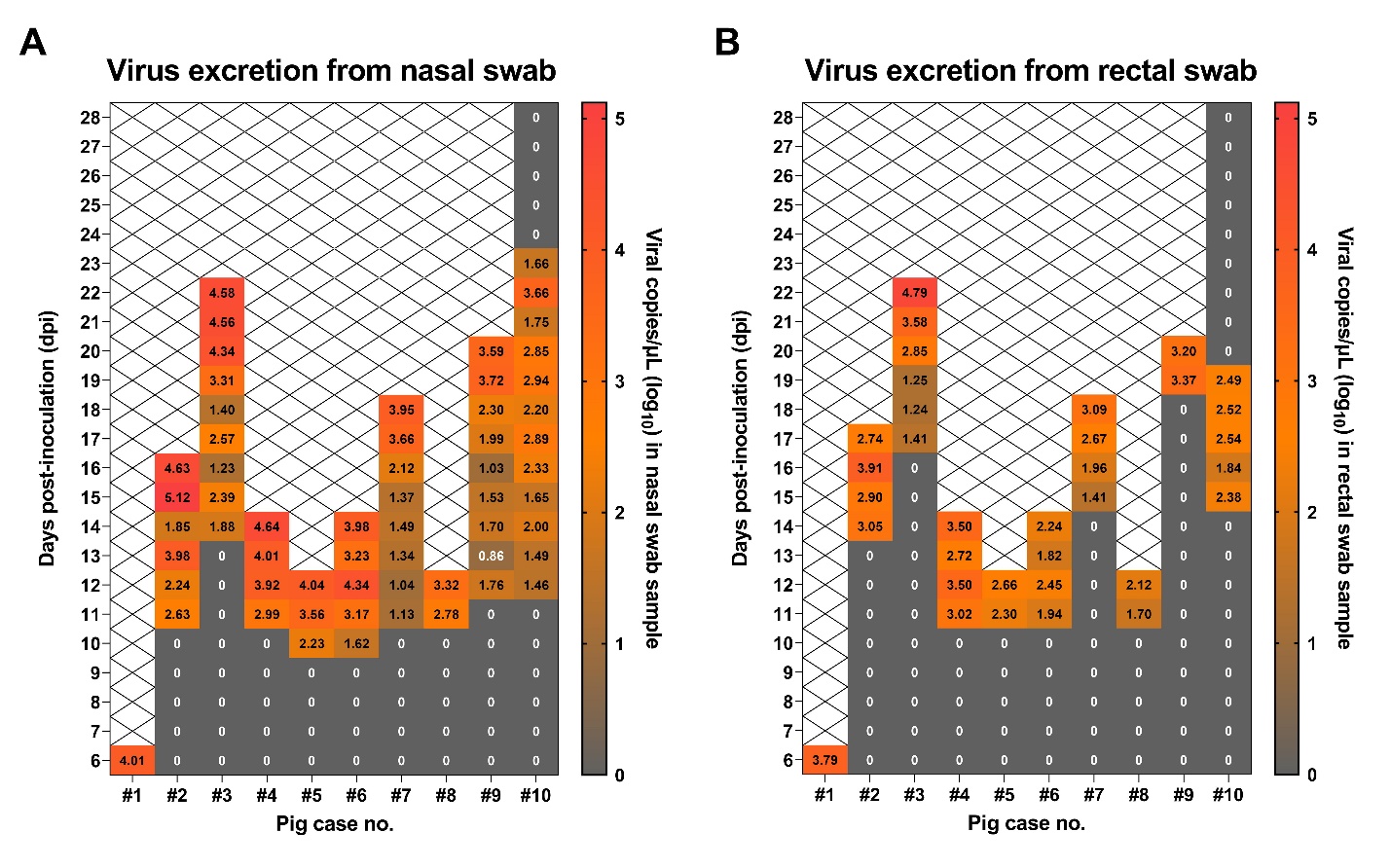
**Supplementary Figure 1.** Histopathological findings in the spleen of experimental animals. Histopathological findings in the spleen from one African swine fever virus-inoculated pig (no. #1), four direct contact-exposed pigs within pen (no. #3, #5, #7, and #10), and one negative control pig (*n* = 1). Pig no. #10 survived during the entire experimental period (28 days-post exposure). (A) Pig no. #1. Severe lymphoid depletion with the presence of neutrophils (arrows) in the marginal zone. (B) Pig no. #3. Mild atrophy of lymphoid follicle. (C) Pig no. #5. Moderate lymphoid depletion with severe engorgement in the red pulp. (D) Pig no. #7. Moderate atrophy of lymphoid follicle with severe engorgement in the red pulp. (E) Pig no. #10. Mild engorgement in the red pulp. (F) Pig no. #15. Lf: lymphoid follicle, Rp: red pulp.


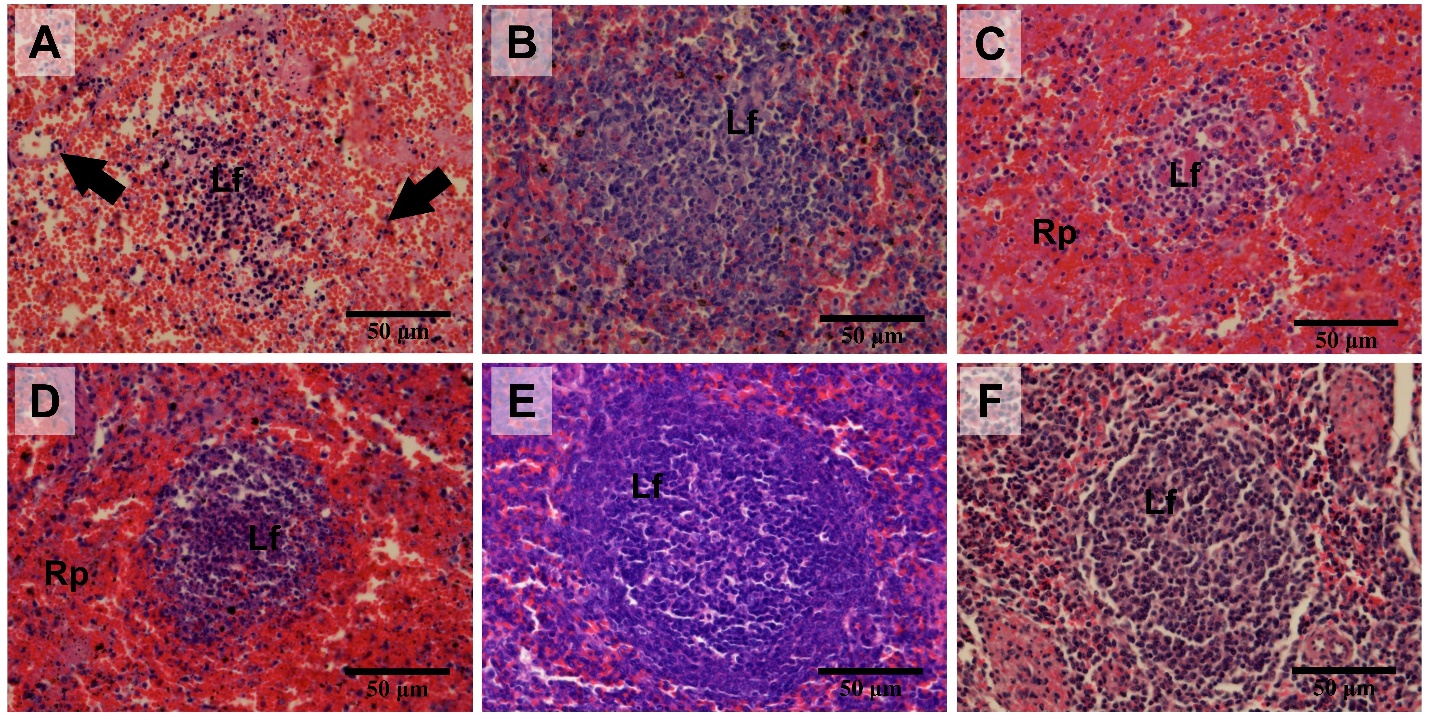
**Supplementary Figure 2.** Patterns of African swine fever virus (ASFV) detection in pigs with experimental pig-to-pig infection. One ASFV-inoculated pig (no. #1) and nine direct contact-exposed pigs within pen (no. #2–#10). (a) Viral copies/μL from individual nasal sample from experimental pigs. (b) Viral copies/μL from individual rectal swab sample from experimental pigs.
